# Symptoms of Patients With Vertebral Artery Dissection Presenting to Chiropractors: A Systematic Review and Meta-Analysis

**DOI:** 10.7759/cureus.51297

**Published:** 2023-12-29

**Authors:** Robert J Trager, Alyssa M Troutner, Harold J Pikus, Clinton J Daniels, Jeffery A Dusek

**Affiliations:** 1 Chiropractic, Connor Whole Health, University Hospitals Cleveland Medical Center, Cleveland, USA; 2 Department of Family Medicine and Community Health, Case Western Reserve University School of Medicine, Cleveland, USA; 3 Department of Biostatistics and Bioinformatics Clinical Research Training Program, Duke University School of Medicine, Durham, USA; 4 Department of Clinical Education, Southern California University of Health Sciences, Whittier, USA; 5 Neurosurgery, Upper Valley Neurology Neurosurgery, Lebanon, USA; 6 Rehabilitation Care Services, Veterans Affairs Puget Sound Health Care System, Tacoma, USA

**Keywords:** systematic review, vertebral artery dissection, transient ischemic attack, spinal manipulation, headache, neck pain, chiropractic, brain infarction

## Abstract

Early symptoms of vertebral artery dissection (VAD) may be nonspecific, including neck pain and headache. Neck pain and headache are also common reasons for patients to seek chiropractic care. We hypothesized that neck pain and/or headache would be the most prevalent symptoms among patients with undiagnosed VAD presenting to chiropractors compared to dizziness or other symptoms. We searched PubMed, Ovid, the Index to Chiropractic Literature, Google Scholar, and gray literature through September 2023 for observational studies describing patients aged ≥10 with previously undiagnosed VAD presenting to a chiropractor. Article selection, data extraction, and quality assessment were performed in duplicate. We synthesized the point prevalence of symptoms and other clinical features. We included 10 case reports describing 10 patients (mean age = 37, SD = 7, 60% female). All patients had either neck pain or headache (100%; 95% confidence interval (CI) = 100%-100%). The most prevalent individual symptoms were neck pain (90%; 95% CI = 71%-100%), headache (80%; 95% CI = 55%-100%), visual disturbance (50%; 95% CI = 19%-81%), and dizziness (40%; 95% CI = 10%-70%). The certainty of results was very low due to publication bias. While our findings suggest that neck pain and/or headache are the most prevalent symptoms among patients with undiagnosed VAD visiting a chiropractor, the small sample size and reliance on case reports preclude any definitive conclusions. Further research with larger sample sizes, control groups, and better control of confounders is required to corroborate these results. Chiropractors should be aware of VAD features and refer suspected patients for emergency care.

## Introduction and background

Paired vertebral arteries ascend through the cervical spine and merge in front of the brainstem to form the basilar artery. The vertebral arteries and basilar artery together are referred to as the vertebrobasilar system, sometimes called the posterior circulation, including the branches to the cerebellum, brainstem, and posterior cerebral distributions [1]. Vertebral artery dissection (VAD) is a tear in the wall of the vertebral artery, leading to the intrusion of blood within its layers [1]. VAD occurs at a median age of 46.5 years [2] and represents a leading cause of stroke in young adults [1], with 63% of cases resulting in a stroke and the remainder resulting in transient ischemic attack (TIA) or subarachnoid hemorrhage [2]. According to a systematic review including 1,972 VAD patients, the most common single symptoms of VAD were dizziness (58% of patients), headache (51%), and neck pain (46%) [2], while 67% of patients had either headache or neck pain [2]. The incidence of VAD is 1 to 1.5 per 100,000 person-years [3].

Many patients with VAD do not seek medical care until they develop neurological symptoms of TIA or stroke. Patients with VAD are relatively young, may have few previously identified comorbidities, and subtle symptoms, and therefore may have an extensive delay between symptom onset and seeking medical care [4]. In one study (n = 41), 15% of patients with VAD who only had neck pain and headache delayed seeking care between nine days and three months [5]. In another study (n = 14), 43% of those with VAD had a delay between headache or neck pain and neurologic dysfunction ranging from one to three weeks [6]. Considering the early, subtle symptoms of VAD, it is plausible some patients would consider their symptoms benign or musculoskeletal in nature and present to a chiropractor.

While neck pain and headache are common symptoms of VAD, these are also common symptoms that prompt patients to seek chiropractic care [7] and are not specific for a diagnosis of VAD [2,5,6]. Neck pain is the second most common reason patients seek chiropractic care after low back pain, with 23% of patients having this complaint [7]. Overall, 7% of chiropractic patients have headaches [7]. In contrast, dizziness is much less common, as chiropractors report only encountering one patient per month with dizziness on average (i.e., <1% of patients) [8].

Previous studies have proposed that a common treatment used by chiropractors, chiropractic spinal manipulation (CSM), may trigger VAD by damaging, occluding, or causing vasospasm or thrombosis in the vertebral arteries [9,10]. However, this hypothesis has been called into question by recent large observational studies which found no increase in the risk of VAD or vertebrobasilar stroke among patients receiving CSM [11-14]. These findings support the idea that neck pain and headache due to undiagnosed VAD may prompt patients to visit a chiropractor, as opposed to CSM causing VAD de novo (i.e., confounding by indication or protopathic bias [11,15]). Additionally, biomechanical studies suggest that the stress/strain of CSM on the vertebral artery is roughly equivalent to activities of daily life and range of motion testing roughly an order of magnitude less than that needed to disrupt the vessel [16-18].

There has been limited examination of the presenting features of patients with undiagnosed VAD who seek chiropractic care. As a primary objective, we report the prevalence of symptoms among patients presenting to chiropractors with undiagnosed VAD. We hypothesized that either neck pain or headache would be the most prevalent presenting symptom, as opposed to dizziness or other symptoms. As a secondary objective, we summarize other demographic and clinical characteristics of these patients.

## Review

Methodology

Study Design

This meta-analysis is structured according to the Preferred Reporting Items for Systematic Reviews and Meta-Analyses (PRISMA) statement [19]. We registered this protocol in the International Prospective Register of Systematic Reviews (PROSPERO: CRD42022319847) and searched PROSPERO and the Cochrane Database of Systematic Reviews to ensure no similar reviews were registered before proceeding. This study was deemed Not Human Subjects Research by the University Hospitals Institutional Review Board (Cleveland, Ohio, USA; STUDY20220370).

Sample Size Calculation

We required at least five patients total among the included studies to allow for a meaningful analysis [20]. This threshold was believed to be feasible given a previous bibliometric review which included multiple cases of VAD presenting to chiropractors [21].

Eligibility Criteria

We included articles reporting patients aged at least 10 [2], with previously undiagnosed VAD, presenting to a chiropractor. For this study, we deemed any clinician with a chiropractic degree (e.g., Doctorate, Bachelors’ or Masters’ degree) to be a chiropractor. We considered clinicians with chiropractic and medical or allied health degree(s) to be chiropractors. Articles describing a diagnosis of VAD confirmed by diagnostic imaging, surgery, or autopsy were included.

Articles describing patients with previously diagnosed VAD were excluded as the association between new and previous clinical features would be unclear. Articles describing patients with carotid artery dissection were included if patients also had VAD. Patients with a diagnosis or evidence of isolated basilar artery dissection, vertebrobasilar insufficiency, vertebral artery pseudoaneurysm, basilar artery migraine, or other cerebrovascular pathology without concomitant VAD were excluded. Animal and cadaver studies were excluded.

Articles describing either a positive outcome or an adverse event following CSM were included provided (1) the presenting symptoms prompting the chiropractic encounter were reported, and (2) pre-existing VAD was confirmed after the chiropractic encounter. The latter could occur if imaging signs of VAD were present, yet not recognized until after CSM was provided [22], or if autopsy findings determined that VAD occurred before CSM [23,24].

Cases appearing in observational studies were included, as these likely provided individual patient data necessary for our study objectives. Experimental studies such as randomized controlled trials were excluded as they reflect protocol-driven care, typically exclude patients with VAD [25,26], and would be unlikely to provide the detail required for our study objectives.

Information Sources

PubMed, Ovid, the Index to Chiropractic Literature, and the first 100 results in Google Scholar (via Harzing’s Publish or Perish Version 8.9) were searched from inception to the search date of July 14, 2023, without language restrictions. Non-English manuscripts were translated using Google Translate. Gray literature searches spanned into September 2023, including reference textbooks of the National Board of Chiropractic Examiners, preprints (medRxiv, The Open Science Framework), theses (ProQuest), review articles [21,27], and asking topic experts for relevant articles. Co-investigators were then allowed to contribute additional references from their personal collection. Reference lists of included articles were hand-searched.

Search Strategy

The search strategy was designed for PubMed by three coauthors (RT, AT, AS), including a research librarian, and adapted for other databases (Appendix A). Keyword lists and search terms from prior publications on the topic of VAD were adapted for the current study [2]. One search theme related to chiropractic, while the other included VAD-related terms such as named vertebrobasilar stroke syndromes and blood vessels and cerebrovascular conditions [1,2]. We used a Boolean “AND” to connect search themes.

Study Records

Study selection was managed with a web interface for systematic reviews (Rayyan, Cambridge, MA, USA). Two reviewers (RT, AT) independently screened titles and abstracts with the aid of a checklist (Appendix B) and independently reviewed full texts. Two reviewers independently extracted data items for each case into a pre-specified Excel workbook (RT, AT, and CD divided this task). All screening and extraction disagreements were resolved through discussion.

Data Items

Data were harmonized according to common terminology [2]. Descriptors of dizziness were recorded in a single category, including vertigo, lightheadedness, and other synonyms [2]. Symptom onset and duration were described categorically [5]. We also recorded demographics, precipitating factors, initial physical examination findings, whether cervical CSM was used, diagnostic testing used to support a VAD diagnosis, and the presence of bony and vascular cranio-cervical variant anatomy. The VAD segment was recorded (i.e., V1, V2, V3, and V4). The result of the VAD was categorized as an ischemic stroke, TIA, or subarachnoid hemorrhage. For patients with infarction, the region(s) of the brain or spinal cord affected was recorded.

Outcomes and Prioritization

Our primary outcome was the point prevalence of patient symptoms, including a composite outcome of neck pain and/or headache, considering these are common reasons for seeking chiropractic care [7]. We compared the point prevalence estimates and 95% confidence intervals (CIs) to determine which symptoms were most common. As a secondary outcome, we reported other patient characteristics.

Risk-of-Bias in Individual Studies

Two raters independently evaluated the reporting quality of included cases using the Joanna Briggs Institute Critical Appraisal Tools Checklist for Case Reports. Scores of 67-100% represented high quality, 34-66% moderate quality, and 0-33% low quality. Discrepancies were resolved through discussion.

Data Synthesis

Clinical variables from included cases were synthesized qualitatively and quantitatively, including a pooled proportion or mean and standard deviation (SD) or 95% CIs for each data item. The article selection process was illustrated using a PRISMA flow diagram [19].

Confidence in Cumulative Evidence

We adapted the Grading of Recommendations, Assessment, Development, and Evaluations (GRADE) approach for grading the certainty of the evidence [28], deeming the domains for “risk of bias” and “publication bias” most applicable to our determination.

Results

Study Selection

**Figure 1:**
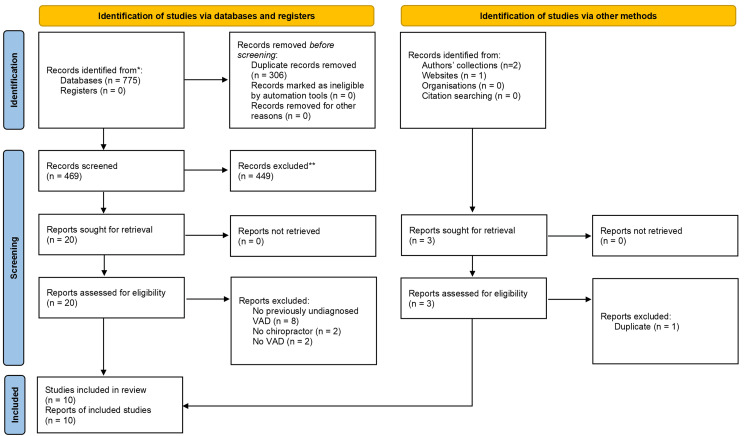
Flow diagram. The selection of studies is illustrated per the Preferred Reporting Items for Systematic Reviews and Meta-Analyses. VAD: vertebral artery dissection

After the removal of duplicates, our database searches identified 469 articles. We identified three additional articles from other sources (Figure 1). Screeners had 96% agreement, and discrepancies were resolved by discussion. We included 10 articles [22-24,29-35]. Reasons for exclusion at full-text screening were no previously undiagnosed VAD (n = 8; [36-43]), no chiropractor (n = 2; [44,45]), no VAD (n = 2; [46,47]), and duplicate article (n = 1; [48]). For two of the articles excluded for lack of previously undiagnosed VAD, it was unclear if the VAD predated the chiropractic encounter [38,41].

Study Characteristics

All included studies were case reports, and each had a single patient included in our analysis. The included articles and their characteristics are described in Table 1.

**Table 1:** Study characteristics. Each included study had one included patient; their clinical features are described in each column. AA: aortic aneurysm; CS: cervical spine; CSM: chiropractic spinal manipulation; CT: computed tomography; ED: emergency department; FHx: family history; F: female; FMD: fibromuscular dysplasia; HA: headache; ↑: increased; IV: intravenous; MRA: magnetic resonance angiography; MRI: magnetic resonance imaging; M: male; mo: month; MVC: motor vehicle collision; NA: not applicable; NR: not reported; ROM: range of motion; TIA: transient ischemic attack; US: ultrasonography; UE: upper extremity; VAH: vertebral artery hypoplasia; wk: week

Author, year	Age; sex; risk factors	Initial symptoms; onset, symptom duration; prior providers	Exam abnormalities; CSM; testing	VA segments; result; brain territory; variant anatomy
Arning et al., 2022 [22]	47; F; NR	Neck pain; acute; NR; NR	NR; yes; MRI, US	2; NR; NR; no variants
Futch et al., 2015 [29]	30; F; migraine; FHx of AA	Neck pain, new type of HA, visual disturbance, eyelid numbness; acute; 1 wk to 1 mo; yes	None; no; MRA	1, 2; TIA; NA; no variants
Giles, 2009 [30]	34; M; smoking; MVC	Neck pain, HA, transient loss of consciousness; gradual; >3 m; yes	↑ symptoms with arm abduction, ↓ CS ROM; no; angiography	Unclear; NR; none; atlas assimilation
Johnson et al., 1993 [23]	44; M; none	Neck and shoulder pain, HA, dizziness, visual disturbance, tinnitus, vomiting, UE weakness; acute; <1 wk; no	NR; yes; autopsy	1, 2; ischemic stroke; cerebellum; VAH
Mas et al., 1989 [24]	35; F; none	Neck pain, HA; NR; 1 wk to 1 mo; no	NR; yes; autopsy	3; ischemic stroke; medulla; no variants
Mattox et al., 2014 [31]	45; F; FMD	Neck pain, HA, dizziness, visual and cognitive disturbances, dysarthria, UE pain; acute; <1 wk; no	↓ CS ROM, CS tenderness; no; CTA, MRA	2; TIA; none; FMD (ICAs)
McCrory, 2000 [32]	27; M; rugby tackle	Neck pain, ataxia, dysarthria, UE pain and paresthesia; acute; NR; yes	NR; unclear; CT, autopsy	Segments unclear; schemic stroke; cerebellum, midbrain, pons; no variants
Mosby et al., 2011 [33]	42; F; self-manipulation	Neck pain, new HA type; worst of life, nausea, vomiting, shoulder pain, visual disturbance; acute; 1 wk to 1 mo; no	CS tenderness; no; MRA	2; NR; none; no variants
Swenson, 1993 [34]	31; M; IV drug use	Occipital HA, nausea, vomiting, dysarthria, dizziness; acute; 1 wk to 1 mo; yes	Eye movement, hyperreflexia, Babinski sign, clonus, sensory deficit; no; angiography, MRI	3; ischemic stroke; cerebellum, occipital lobes, midbrain; no variants
Tarola et al., 2015 [35]	34; F; smoking	Neck pain, HA, dizziness, visual and auditory disturbance, UE paresthesia, ataxia; acute; <1 wk; no	↓ CS ROM, CS tenderness, edema right upper CS; no; MRA	1, 2; TIA; none; VAH

Article Quality

Article quality was high in nine articles (90%) [23,24,29-35] and moderate in one (10%) [22] (Table 2).

**Table 2:** Article quality. Numbers correspond to the items in the Joanna Briggs Institute Critical Appraisal Tools Checklist for Case Reports which can be summarized as (1) demographics, (2) history/timeline, (3) current clinical condition, (4) diagnostic tests, (5) intervention/treatment, (6) post-intervention condition, (7) adverse events, and (8) takeaway lessons. Y: yes (green); N: no (red); U: unclear (orange); NA: not applicable (white)

Author, year	1	2	3	4	5	6	7	8	Quality
Arning et al., 2022 [22]	Y	N	U	Y	N	N	Y	Y	Moderate
Futch et al., 2015 [29]	Y	Y	Y	Y	Y	Y	NA	Y	High
Giles, 2009 [30]	Y	Y	Y	Y	Y	Y	NA	Y	High
Johnson et al., 1993 [23]	Y	Y	U	Y	Y	Y	Y	Y	High
Mas et al., 1989 [24]	Y	Y	Y	Y	Y	Y	Y	Y	High
Mattox et al., 2014 [31]	Y	Y	Y	Y	Y	Y	NA	Y	High
McCrory, 2000 [32]	Y	N	Y	Y	Y	Y	U	Y	High
Mosby et al., 2011 [33]	Y	Y	Y	Y	Y	Y	NA	Y	High
Swenson, 1993 [34]	Y	Y	Y	Y	Y	Y	NA	Y	High
Tarola et al., 2015 [35]	Y	Y	Y	Y	Y	Y	NA	Y	High

Results of Syntheses

**Figure 2:**
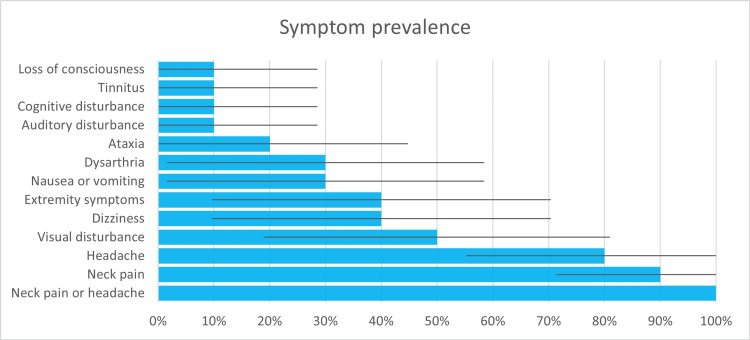
Point prevalence of symptoms among patients with vertebral artery dissection presenting to chiropractors. Error bars indicate 95% confidence intervals.

For our primary outcome of symptom point prevalence, all 10 patients had either neck pain or headache (100%, 95% CI = 100%-100%) (Figure 2). For individual symptoms, the prevalence was greatest for neck pain (90%; 95% CI = 71%-100%), followed by headache (80%; 95% CI = 55%-100%), visual disturbance (50%; 95% CI = 19%-81%), and dizziness (40%; 95% CI = 10%-70%), with other symptoms being less common. Headache was described as a new type of headache and/or the worst headache of life in two cases [29,33].

Of the 10 included patients, the mean age was 36.9 years (SD = 6.7; range = 27-47) and six were female (Table 1). Risk factors for VAD were reported in seven patients, including smoking [30,35], trauma [30,32], fibromuscular dysplasia [31], history of migraine [29], family history of aortic aneurysm [29], and intravenous drug use [34]. Symptom onset was abrupt in eight patients yet varied in duration ranging from less than one week to more than three months (Table 1). Four patients were reported to have seen other providers before the chiropractic encounter.

Examination findings were not reported in four of the 10 patients. Only three patients had relevant abnormalities reported, one of which included neurological and cranial nerve findings [34] (Table 1). CSM was administered in only three patients, not administered in six, and not reported if administered in one. Diagnostic testing varied, including two patients in which an autopsy detected and characterized the VAD. VAD was reported to affect segments one through three (i.e., V1, V2, V3) in eight patients while the specific segment was not reported in two. Four patients suffered an ischemic stroke, three suffered a TIA, and the result was not reported in the remaining three. The cerebellum was the region affected in three of the four patients suffering ischemic stroke. Variant anatomy described included vertebral artery hypoplasia in two patients, atlas assimilation in one patient, fibromuscular dysplasia in another patient, and was not reported or absent in the remaining six patients.

Heterogeneity

Considering we included 10 case reports, there was no within-study variance to account for, and thus we did not calculate an I^2^ value. We did not perform any sensitivity analyses or subgroup analyses considering the small sample size.

Certainty of Evidence

We downgraded the certainty of evidence by three levels to very low due to the limited number of cases synthesized and the inherent risk of publication bias evident in case reports [20]. We did not downgrade due to indirectness as synthesized outcomes aligned with those of interest. We did not downgrade due to quality (risk of bias) which was moderate or high for all studies.

Discussion

The results of this meta-analysis offer tentative support for our hypothesis that patients visiting a chiropractor with undiagnosed VAD have a higher prevalence of neck pain and/or headache compared to dizziness or other symptoms. Our findings, therefore, support the notion that patients experiencing symptoms related to evolving VAD may inadvertently visit a chiropractor due to concurrent neck pain and/or headache, potentially confounding the association between CSM and VAD.

Our results give some insights into the overall clinical features of patients with evolving VAD that present to chiropractors. Patients were young to middle-aged adults and most often had an abrupt onset of symptoms, which are expected features of VAD [2,5]. While neck pain, headache, dizziness, and visual disturbances were relatively common, the risk factors, duration of symptoms, and examination findings varied. The examination was often noncontributory toward diagnosis, with only one case reporting detailed neurological findings [34], while in another case, VAD was suspected based on the patient’s history alone [29]. In one case, fibromuscular dysplasia was only identified after the VAD was diagnosed (i.e., the chiropractor had no prior knowledge of this risk factor) [31]. Our findings are, therefore, consistent with the notion that VAD may be challenging to identify based on its clinical features alone [4].

Chiropractors should be aware of the risk factors and presenting signs and symptoms of VAD. The median age of a chiropractic patient is 43, which is similar to the typical age for VAD in the general population and in the present study sample [7]. Apart from neck pain and headache being two of the most common reasons for seeking chiropractic care [7], chiropractors routinely encounter those at risk for VAD, such as pregnant women and patients with hypertension [7,49]. Patients with hypermobility syndromes (i.e., connective tissue disorders) frequently have musculoskeletal pain and may seek chiropractic care, yet are at an increased risk of spontaneous VAD [2,50]. Patients with other forms of vasculopathy due to infectious, inflammatory, autoimmune, and genetic diseases can be at a higher risk for spontaneous VAD as well [50].

VAD is a difficult diagnosis to make and requires advanced vascular imaging considering there are no specific or reliable bedside tests for this condition [2,51-53]. Neck pain and headache are highly prevalent in general and in the VAD population, yet nonspecific for VAD in isolation [2,5,6]. In addition, the incidence of VAD is low [54], therefore, it is impractical and potentially harmful to obtain advanced vascular imaging for all patients presenting with only neck pain and headaches. Vascular imaging can be costly, have limited availability, may unnecessarily expose patients to contrast agents with risk of allergy [51], could reveal incidental findings that lead to patient anxiety without providing clinical benefit, and may delay necessary care if patients are required to undergo testing before treatment for their pain. Accordingly, VAD is frequently missed in a range of healthcare settings [2,4,52]. Considering the consequences of missed VAD are potentially catastrophic, there is a need to better understand which patients should merit suspicion. Improved methods of identifying VAD in a clinical setting are desperately needed.

Our findings should be corroborated by larger study designs using records-based or claims-based datasets. A case-control design could be used to compare the prevalence of preceding headache and neck pain between individuals with VAD who visited a chiropractor versus a primary care clinician using logistic regression to account for confounding variables. A greater prevalence of neck pain and/or headache among individuals presenting to chiropractors relative to primary care clinicians would support the hypothesis that such symptoms could prompt patients with VAD to visit a chiropractor. In addition, a case-control design could be used to identify the odds of visiting a chiropractor versus other clinician types given the presence of headache and/or neck pain among individuals later developing VAD.

Our work highlights the need for better interdisciplinary investigation into the topic of VAD and chiropractic. Cases describing VAD potentially associated with CSM provide the most actionable information when they describe the patient’s symptoms and examination findings before CSM at the time of the chiropractic encounter, as well as the type of CSM performed and the patient’s response, yet these details are often lacking [27]. Advanced imaging techniques, such as stroke-protocol MRI and perfusion imaging, allow us to date ischemic stroke [55]. Similar techniques can be used to time the onset of the dissection itself and could better clarify the nature of the temporal association between CSM, VAD, and stroke. We, therefore, call for greater collaborative efforts in reporting pre-existing symptoms and detailing the chiropractic management strategy as well as comprehensive imaging of the involved vessels and brain in cases reporting VAD following CSM.

Strengths and limitations

The methodological strengths of this review include adherence to a registered protocol developed by an interdisciplinary team, a comprehensive search strategy without language restrictions, inclusion of gray literature, and duplicate article selection and data extraction. However, some important limitations should be considered. This study lacks a control group, and we relied on external epidemiologic comparisons [2], precluding any direct comparison to patients with evolving VAD visiting primary care clinicians. For this reason, we were also unable to calculate a measure of likelihood (e.g., risk ratio, odds ratio) for patients to visit a chiropractor versus a primary care clinician. This study was based on a limited number of patients (i.e., 10), thus yielding wide imprecise estimates for the point prevalence of neck pain, headache, dizziness, and other symptoms. All patients had either neck pain or headache (no variability), leading to an inability to calculate a meaningful 95% CI for this composite outcome. Our findings were solely based on case reports, which have an inherent publication bias due to the potential for selective reporting or focus on atypical presentations. Additionally, case reports rely on patient-reported symptoms, potentially leading to inaccuracies in the medical history and risk factors. Our secondary outcomes based on imaging findings should be interpreted with caution in older cases given radiological advancements in recent years [51]. We were unable to estimate the incidence of VAD presenting to chiropractors based on case reports. Due to our focus on chiropractors, we were unable to determine whether our findings generalize to other types of clinicians who may also use manual therapies to treat neck pain and headaches, such as physical therapists, osteopaths, or massage therapists. Overall, the small sample size, reliance on case reports, and potential publication bias necessitate further research with larger sample sizes, control groups, and better control of confounders. Addressing these limitations would enhance the certainty of the findings, ultimately contributing to improved patient care.

## Conclusions

The findings from this meta-analysis provide very low certainty evidence that individuals with in-progress VAD who visit a chiropractor have a high prevalence of neck pain and/or headache compared to other symptoms. This finding should be interpreted with caution due to the small sample size and potential publication bias, which prohibit drawing any firm conclusions. Additional research is needed to corroborate our findings, for example, using a case-control design to examine the odds of preceding headache and/or neck pain among individuals with VAD who visited a chiropractor versus a primary care clinician. Considering neck pain and headache alone are insufficiently specific to merit either abstention from chiropractic care or justify workup for VAD, chiropractors should be vigilant to identify additional clinical features and risk factors for VAD such as pregnancy, hypermobility, and vasculopathy due to infectious, inflammatory, autoimmune, and genetic diseases. Chiropractors should avoid CSM in any patients with a high risk of concurrent VAD and refer them for emergent medical attention.

## Appendices

Appendix A

**Table 3:** Search strategies.

Database (number of results)	Search string
Pubmed (345)	(Chiropractic[mesh] OR “manipulation, chiropractic”[mesh] OR Chiropract*[tiab]) AND (Anterior spinal artery syndrome[mesh] OR Anterior spinal arter*[tiab] OR Anton syndrom*[tiab] OR Antons syndrom*[tiab] OR Anton’s syndrom*[tiab] OR Arterial dissection*[tiab] OR Artery dissection*[tiab] OR Basilar artery[mesh] OR Basilar arter*[tiab] OR Basilar syndrom*[tiab] OR Beauty parlor stroke*[tiab] OR Beauty parlour stroke*[tiab] OR Benedikt syndrom*[tiab] OR Benedikt’s syndrom*[tiab] OR Benedict syndrom*[tiab] OR Benedict’s syndrom*[tiab] OR “Blindness, cortical”[mesh] OR Brain stem infarction[mesh] OR Cerebrovascular accident*[tiab] OR Claude syndrom*[tiab] OR Claude’s syndrom*[tiab] OR Cortical blindness[tiab] OR Dejerine syndrom*[tiab] OR Dejerine’s syndrom*[tiab] OR Dejerine-roussy syndrom*[tiab] OR Drop attack*[tiab] OR Foville syndrom*[tiab] OR Lateral medullary syndrome[mesh] OR Lateral pontine syndrom*[tiab] OR “locked-in syndrome”[mesh] OR Marie foix alajouanine syndrom*[tiab] OR Marie foix syndrom*[tiab] OR Medullary syndrom*[tiab] OR Millard gublar syndrom*[tiab] OR Millard gubler syndrom*[tiab] OR “one and a half syndrom*”[tiab] OR Pontine syndrom*[tiab] OR Posterior cerebral ischemia[tiab] OR Posterior circulation*[tiab] OR Raymond syndrom*[tiab] OR Stroke[mesh] OR Stroke*[tiab] OR Vertebral artery[mesh] OR Vertebral artery dissection[mesh] OR Vertebral arter*[tiab] OR Vertebral basilar[tiab] OR Vertebrobasilar[tiab] OR Wallenberg syndrom*[tiab] OR Wallenberg’s syndrom*[tiab] OR Weber syndrom*[tiab] OR Weber’s syndrom*[tiab])
OVID (214)	((exp Chiropractic/ OR “manipulation, chiropractic”.mp. OR Chiropract*.ti.) AND (exp Anterior Spinal Artery Syndrome/ OR “Anterior spinal arter*”.ti. OR “Anton syndrom*”.ti. OR “Antons syndrom*”.ti. OR “Anton's syndrom*”.ti. OR “Arterial dissection*”.ti. OR “Artery dissection*”.ti. OR exp Basilar Artery/ OR “Basilar arter*”.ti. OR “Basilar syndrom*”.ti. OR “Beauty parlor stroke*”.ti. OR “Beauty parlour stroke*”.ti. OR “Benedikt syndrom*”.ti. OR “Benedikt’s syndrom*”.ti. OR “Benedict syndrom*”.ti. OR “Benedict’s syndrom*”.ti. OR “Blindness, cortical”/ OR exp Brain Stem Infarctions/ OR “Cerebrovascular accident*”.ti. OR “Claude syndrom*”.ti. OR “Claude’s syndrom*”.ti. OR “Cortical blindness”.ti. OR “Dejerine syndrom*”.ti. OR “Dejerine’s syndrom*”.ti. OR “Dejerine-Roussy syndrom*”.ti. OR “Drop attack*”.ti. OR “Foville syndrom*”.ti. OR exp Lateral Medullary Syndrome/ OR “Lateral pontine syndrom*”.ti. OR “locked-in syndrome”/ OR “Marie Foix Alajouanine syndrom*”.ti. OR “Marie Foix syndrom*”.ti. OR “Medullary syndrom*”.ti. OR “Millard Gublar syndrom*”.ti. OR “Millard Gubler syndrom*”.ti. OR “one and a half syndrom*”.ti. OR “Pontine syndrom*”.ti. OR “Posterior cerebral ischemia”.ti. OR “Posterior circulation*”.ti. OR “Raymond syndrom*”.ti. OR (exp Stroke/ OR Stroke*.ti.) OR exp Vertebral Artery/ OR exp Vertebral Artery Dissection/ OR “Vertebral arter*”.ti. OR “Vertebral basilar”.ti. OR “Vertebrobasilar”.ti. OR “Wallenberg syndrom*”.ti. OR “Wallenberg’s syndrom*”.ti. OR “Weber syndrom*”.ti. OR “Weber’s syndrom*”.ti.))
Index to Chiropractic Literature (116)	All Fields:\\\“vertebral artery\\\”
Google Scholar (100)	Chiropractic AND (Artery dissection OR Basilar artery OR Infarction OR Cerebrovascular accident OR Drop attack OR Cerebral ischemia OR Posterior circulation OR Stroke OR Vertebral artery OR Vertebral basilar OR Vertebrobasilar)

Appendix B

**Table 4:** Screening checklist.

Criteria	Yes	No	Unclear
Patient age ≥10	☐	☐	☐
Previously undiagnosed vertebral artery dissection	☐	☐	☐
Patient presenting to a chiropractor	☐	☐	☐
Presenting symptoms at chiropractic encounter reported	☐	☐	☐
Observational study (e.g., case report, series, chart review, cohort)	☐	☐	☐
